# The Sufferings of Christ

**Published:** 1887-08-20

**Authors:** 


### Words of Consolation.

[Communications for this column should be addressed, "Chaplain," care of the Editor of The Hospital, who will gratefully welcome any suggestions or inquiries from matrons, nurses, and the staff generally.]

### XLVI.—The Sufferings of Christ.

(*For reading to the Sick.*)

Further on we shall try and enter more fully into the details of our Blessed Lord's sufferings. For the present let us briefly glance at the outline of His Passion from Gethsemane to the great darkness of His last agony on the Cross. Picture Him to yourself, first of all bowed down prostrate on His face in the Garden of Gethsemane, sweating great drops of blood for the very anguish of His soul. See Him here convulsed with fear and amazement, violently agitated by some mysterious and appalling revelation of sin and its author which caused Him to lift up His voice "with strong crying and tears." "My soul is exceeding sorrowful, even unto death: . . . O my Father, if it be possible, let this cup pass from me: nevertheless not as I will, but as thou wilt" (Matt xxvi, 38, 39). Whatever assaults He experienced here from the malignity of the Evil One, and from other wicked spirits—for this was their hour and the power of darkness—He must, besides, have become anxious of His office as the Sin-bearer, to a degree unknown to Him before. The feeling that He, being innocent, had to endure the sense of the curse due unto sin, was overwhelming His guileless soul. It is hard to suffer patiently, sometimes, what you know you deserve; but if you have ever had to suffer for what you did not deserve, you know that was harder still. In such a case you have thought yourself entitled to sympathy indeed. Try, then, and feel a little more deeply than you have yet for Him. Pray that you may be sufficiently awakened to a sense of the hideous nature of sin, to watch with Him one hour on that awful night of His agony. Watch Him attentively with the weight of your guilt pressing with relentless force upon His innocent soul. Mark, for one moment, that He shed tears there for you, that He there began to bleed for you. Then add to this the rest of the cup He drank, keeping, for the present, your own case in view as *the cause*. Consider the betrayal by the traitor, the falsest of friends; the false accusations; the mocking; the insults; the spitting; the scourging; the crown of thorns; His sacred figure continually streaming down with blood; His visage more marred than any man's; His limbs quivering under the scourges; His body exposed to every variety of torture wickedness could devise, even before He set out on the way to Calvary. Behold Him enduring all these things in perfect patience, for the most part in silence, for your sake. Follow Him thence, led like a lamb to the slaughter, bearing His Cross until He broke down under the weight of the beams; then submitting to be nailed to the tree of shame; praying for you while the nails were driven into His hands and feet; willing to hang there for hours, to hear Himself reviled while dying to save His enemies; suffering at last a depth of woe in the darkness into which we can never penetrate. Only this we know—such was the darkness, the utter desolation experienced within His soul in bearing the curse due unto sin, that He felt as if the very presence of His Father was withdrawn, and cried with a loud voice, "My God, My God, why hast Thou forsaken Me?" (Matt. xxvii, 46).

(*To be continued.*)

